# Pre-diagnostic prescription patterns in bladder and renal cancer: a longitudinal linked data study

**DOI:** 10.3399/BJGP.2023.0122

**Published:** 2023-12-12

**Authors:** Garth Funston, Marie Moullet, Luke Mounce, Georgios Lyratzopoulos, Fiona M Walter, Yin Zhou

**Affiliations:** Department of Public Health and Primary Care, University of Cambridge, Cambridge; Wolfson Institute of Population Health, Queen Mary University of London, London.; Wolfson Institute of Population Health, Queen Mary University of London, London.; University of Exeter Medical School, University of Exeter, Exeter.; Insititute of Epidemiology and Healthcare, University College London, London.; Wolfson Institute of Population Health, Queen Mary University of London, London.; Department of Public Health and Primary Care, University of Cambridge, Cambridge; Wolfson Institute of Population Health, Queen Mary University of London, London.

**Keywords:** urinary tract infections, bladder cancer, primary health care, kidney cancer, atrophic vaginitis, renal cell cancer

## Abstract

**Background:**

Understanding pre-diagnostic prescribing activity could reveal windows during which more timely cancer investigation and detection may occur.

**Aim:**

To examine prescription patterns for common urological clinical features prior to renal and bladder cancer diagnoses.

**Design and setting:**

A retrospective cohort study was performed using electronic primary care and cancer registry data on patients with bladder and renal cancer, who received their diagnosis between April 2012 and December 2015 in England.

**Method:**

Primary care prescriptions up to 2 years pre- diagnosis were analysed for five groups of clinical features (irritative urological symptoms, obstructive symptoms, urinary tract infections [UTIs], genital infections, and atrophic vaginitis). Poisson regressions estimating the inflection point from which the rate of prescriptions increased from baseline were used to identify the start of diagnostic windows during which cancer could be detected.

**Results:**

A total of 48 094 prescriptions for 5322 patients were analysed. Inflection points for an increase in UTI prescriptions were identified 9 months pre- diagnosis for renal (95% confidence interval [CI] = 5.3 to 12.7) and bladder (95% CI = 7.4 to 10.6) cancers. For bladder cancer, the change in UTI antibiotic prescription rates occurred 4 months earlier in females (11 months pre- diagnosis, 95% CI = 9.7 to 12.3) than in males (7 months pre-diagnosis, 95% CI = 5.4 to 8.6). For other clinical features, no inflection points were identified and, as such, no diagnostic windows could be defined.

**Conclusion:**

Prescription rates for UTIs increased 9 months before bladder and renal cancer diagnoses, indicating that there is potential to expedite diagnosis of these cancers in patients presenting with features of UTI. The greatest opportunity for more timely diagnosis may be in females with bladder cancer, who experienced the earliest increase in UTI prescription rate.

## INTRODUCTION

In the UK, around 13 000 and 10 000 people are diagnosed with renal and bladder cancer, respectively, each year.^1^^,^^2^ The majority are diagnosed after they present with urological symptoms in general practice and are, subsequently, referred for specialist investigation.^3^ Patients with bladder and renal cancer frequently present to primary care multiple times before diagnosis, and studies have identified the presence of potential missed opportunities for timely detection.^4^^–^6

Clinical activities, such as primary care consultation rates and routine tests, can increase in patients many months prior to cancer diagnosis. These periods, particularly if prolonged, may represent ‘windows’ in which cancer can be detected earlier in some patients.^7^ Understanding when and how clinical activities change during this period can help to determine the potential for more timely diagnosis.

Studies on colorectal cancer, Hodgkin lymphoma, lung cancer, and all cancers have demonstrated increased rates of prescriptions pre-diagnosis, both in terms of certain specific medications and when all prescriptions were examined collectively.^8^^–^12 The authors of the study presented here have previously examined pre-diagnostic primary care blood tests and secondary care imaging test patterns in patients with bladder and renal cancer, but are not aware of any studies exploring prescription patterns prior to the diagnoses of these malignancies.^13^^,^^14^ In this study, patterns of relevant primary care prescriptions for urological symptoms and conditions in the 24 months before renal and bladder cancer diagnoses were examined. The authors focused on medications used to treat symptoms (for example, urgent or irritative urological symptoms) or conditions (for example, urinary tract infections [UTIs]), which may be a manifestation or concomitant feature of urological cancer. The authors aimed to identify inflection points — that is, time points from which changes in the rate of prescribing occurred — in order to define diagnostic windows during which opportunities to detect cancer earlier might exist.

**Table:** How this fits in

Previous studies have demonstrated that prescription rates for certain medications increase many months before the diagnosis of some cancers. Determining whether prescribing for common urological clinical features increases in patients with renal and bladder cancer could help to indicate opportunities for more timely diagnosis. This study found that prescription rates for medications for urinary tract infections (UTIs) increased 9 months before bladder and renal cancer diagnosis, with an even earlier increase occurring before bladder cancer diagnosis in females (11 months). This indicates that there is a window of opportunity during which investigation and referral could lead to earlier cancer detection in some patients presenting to their GP with features of UTI.

## METHOD

### Study design

A retrospective cohort study was performed using linked data from the Clinical Practice Research Datalink (CPRD) GOLD database and cancer registry data, consisting of 5322 patients with bladder, renal, and upper tract urothelial cancer (UTUC), who were diagnosed between April 2012 and December 2015 in England. UTUC was distinguished from the other two cancers as it arises mostly from the renal pelvis, but presents more similarly to bladder cancer. Its distinction is to allow more reliable interpretation of the findings of the bladder and renal cancer cohorts, as the overall number of UTUC cases was small.

The study cohort was a subset derived from a linked dataset containing patients with 11 common cancers identified via cancer diagnosis codes in the CPRD. Patients were aged ≥25 years, with a first diagnosis of these cancers. This cohort has been described previously.^13^

### Prescription categories

The authors identified prescriptions recorded in the CPRD GOLD database in the 24 months pre-diagnosis, as such changes have previously been noted as early as 18 months prior to cancer diagnosis.^7^ Included prescriptions were those for medications used to treat symptoms that might be related to renal and bladder cancer, or to conditions that might mimic these cancers. These include irritative urinary symptoms (such as dysuria, urinary frequency, and urgency) and obstructive urinary symptoms (such as, hesitancy, poor stream, and urinary retention). Also included were prescriptions used to treat UTIs, genitourinary infections, and atrophic vaginitis, as there is a significant overlap between the symptom profiles of these conditions and those of renal and bladder cancer.

Medication categories were defined in line with *British National Formulary* (BNF) treatment categories for five groups of clinical features (Box 1).^15^ Two of the authors reviewed all medication lists to ensure relevance (see Supplementary Table S1).

**Box 1.** Medication categories and rationale for inclusion

**Clinical feature examined**	**Rationale**		**Medication category in *British National Formulary*, and examples^15^**	**Prescription dimension studied**	
	
	**A^a^**	**B^b^**		**Rate of prescription**	**Rate of first prescription**
Irritative symptoms	X		**Antimuscarinics** — oxybutynin hydrochloride, immediate-release tolterodine tartrate, or darifenacin	X	X

Obstructive symptoms	X		**Alpha-blockers** — for example, tamsulosin, doxazosin, and alfuzosin	X	X
			**Beta-agonists**		
			**Alpha-reductase** — for example, finasteride		

Urinary tract infection		X	**Antibiotics** listed in *British National Formulary* as a treatment for urinary tract infection or pyelonephritis — for example, nitrofurantoin and trimethoprim	X	
			**Alkalysing agents** used in the treatment of cystitis		

Genital infections		X	**Antifungals** and **antibacterial** topical treatments	X	

Atrophic vaginitis		X	**Topical oestrogens**	X	X

a *Symptoms related to bladder and renal cancer, or to conditions that might mimic these cancers.*

b *Conditions with symptoms that overlap with those of bladder and/or renal cancer.*

### Analysis

A descriptive analysis was performed summarising:

- frequency and rate of prescription by clinical features; and
- frequency of first prescriptions for three clinical features (namely, irritative symptoms, obstructive symptoms, and atrophic vaginitis) by examining historical prescription records.

The latter features often have a more gradual presentation and chronic history of progression, such as in urinary incontinence, benign prostatic disease, or atrophic vaginitis; as such, an entirely new prescription type could signify the onset of a new symptom. As in previous studies, prescriptions issued in the month before diagnosis were excluded from the authors’ analyses, due to the likelihood that the patients to whom they had been given would have already entered the final stage of the diagnostic process for cancer.^13^

Next, a series of multi-level Poisson regression models were constructed, with adjustments for age, to identify the most likely month (defined as a 28-day period, resulting in 26 such ‘periods’ over 2 years) when cohort-level rates of prescriptions increased above baseline. The models were based on the concept of joinpoint regression, which examined discontinuity in trends.^16^^,^^17^ This approach has been used in the authors’ previous studies^13^^,^^14^^,^^18^ examining pre- diagnostic patterns of healthcare events. Each model included a variable to account for the background rate, which increased from 0 at 2 years pre-diagnosis to 24 in the period before diagnosis. Separate ‘inflection month’ variables captured any deviation from the background trend, with separate models for each possible month of inflection. This inflection variable was held at 0 for all months prior to the inflection month for that model and then increased by one for each month up to diagnosis. The model with the largest log likelihood was considered the best-fitting model, and the corresponding month for its included inflection point was noted; 95% confidence intervals (CIs) for this month were then estimated via bootstrapping. Inflection points were discounted when 95% CIs spanned either the diagnosis date or the study start date (2 years pre-diagnosis). A detailed explanation of the method has been published elsewhere.^19^

Bladder cancer, renal cancer, and UTUC were considered separately in the analysis because they can present differently and, therefore, associated prescriptions may differ. Inflection points were estimated for all combined prescriptions for each clinical feature (for example, antibiotics and alkalysing agents collectively for UTI) and medication category (that is, antibiotics and alkalysing agents separately), as grouped in Box 1. Given prior evidence on the potential sex inequality and contribution of UTI in the timeliness of bladder cancer diagnosis,^4^^–^6^,^^20^^,^^21^ rates of prescriptions were described separately for males and females, and a further analysis examining the inflection point for UTI prescriptions was performed by sex.

All analyses were performed in Stata/IC (version 16.1). Graphs were drawn using R and the ggplot package.

## RESULTS

In total, 5322 patients with linked CPRD and cancer registry data were included. A total of 48 094 prescriptions up to 2 years pre- diagnosis from patients with bladder cancer (*n* = 3398, 63.8%), renal cancer (*n* = 1715, 32.2%), and UTUC (*n* = 209, 3.9%) were examined; prescription data are shown in Table 1.

**Table 1.** Patient characteristics by sex and by cancer site for bladder cancer (*n* = 3398), renal cancer (*n* = 1715), upper tract urothelial cancer (UTUC), (*n* = 209), and overall (*n* = 5322)

**Characteristic, *n* (%)**	**Bladder cancer**		**Renal cancer**		**UTUC**		**Overall**	

	**Female**	**Male**	**Female**	**Male**	**Female**	**Male**	**Female**	**Male**
**Total**	941	2457	649	1066	88	121	1678	3644

**Age group, years^a^**								
<35	4 (0.4)	7 (0.3)	7 (1.1)	10 (0.9)	0 (0.0)	0 (0.0)	11 (0.7)	17 (0.5)
35–44	22 (2.3)	37 (1.5)	34 (5.2)	39 (3.7)	1 (1.1)	2 (1.7)	57 (3.4)	78 (2.1)
45–54	59 (6.3)	130 (5.3)	76 (11.7)	151 (14.2)	3 (3.4)	15 (12.4)	138 (8.2)	296 (8.1)
55–64	135 (14.3)	367 (14.9)	127 (19.6)	240 (22.5)	17 (19.3)	18 (14.9)	279 (16.6)	625 (17.2)
65–74	281 (29.9)	801 (32.6)	189 (29.1)	326 (30.6)	30 (34.1)	44 (36.4)	500 (29.8)	1171 (32.1)
75–84	298 (31.7)	810 (33.0)	137 (21.1)	225 (21.1)	27 (30.7)	29 (24.0)	462 (27.5)	1064 (29.2)
≥85	142 (15.1)	305 (12.4)	79 (12.2)	75 (7.0)	10 (11.4)	13 (10.7)	231 (13.8)	393 (10.8)

**Prescriptions given up to 2 years pre-diagnosis**	33 421		12 881		1792		48 094	
Total^b^	7136 (21.4)	26 285 (78.6)	3471 (26.9)	9410 (73.1)	716 (40.0)	1076 (60.0)	11 323 (23.5)	36 771 (76.5)
Per patient	7.8	10.7	5.3	8.8	3.4	8.9	6.7	10.1

**Relevant prescriptions (by clinical feature)**	20 704		7759		1126		29 265	
Total by cancer/sex^c^	5377 (75.4)	15 327 (58.3)	2422 (69.8)	5337 (56.7)	491 (68.6)	635 (59.0)	7966 (70.4)	21 299 (57.9)
Prescription per patient	5.7	6.2	3.7	5.0	5.6	5.2	4.7	5.8
Irritative symptoms	1117 (20.8)	1148 (7.5)	474 (19.6)	484 (9.1)	185 (37.7)	33 (5.2)	1776 (22.3)	1665 (7.8)
Obstructive symptoms	658 (12.2)	9991 (65.2)	575 (23.7)	3635 (68.1)	40 (8.1)	417 (65.7)	1273 (16.0)	14 043 (65.9)
Urinary tract infection	3261 (60.6)	4080 (26.6)	1251 (51.7)	1176 (22.0)	241 (49.1)	180 (28.3)	4753 (59.7)	5436 (25.5)
Atrophic vaginitis	116 (2.2)	0 (0.0)	41 (1.7)	0 (0.0)	7 (1.4)	0 (0.0)	164 (2.1)	0 (0.0)
Genital infections	225 (4.2)	108 (0.7)	81 (3.3)	42 (0.8)	18 (3.7)	5 (0.8)	324 (4.1)	155 (0.7)

**First prescription^a^**								
For at least one of the three features below	440 (46.8)	1224 (49.8)	68 (10.5)	356 (33.4)	25 (28.4)	36 (29.8)	533 (31.8)	1616 (44.3)
Irritative symptoms	375 (39.9)	464 (18.9)	52 (8.0)	108 (10.1)	15 (17.0)	18 (14.9)	442 (26.3)	590 (16.2)
Obstructive symptoms	85 (9.0)	1132 (46.1)	24 (3.7)	336 (31.5)	15 (17.0)	24 (19.8)	124 (7.4)	1492 (40.9)
Atrophic vaginitis	155 (16.5)	0 (0.0)	12 (1.8)	0 (0.0)	5 (5.7)	0 (0.0)	172 (10.3)	0 (0.0)

a *Percentages represent the proportion of patients in this category with a corresponding prescription.*

b *Percentages represent the proportion of total prescriptions for each cancer attributed to male and female patients.*

c *Percentages represent the proportion of total relevant prescriptions for each cancer attributed to male and female patients.*

### Prescription for different clinical features

Between 60% and 63% of all prescriptions were for the five examined clinical features (that is, relevant prescriptions; data not shown). The average number of relevant prescriptions in the 2 years pre-diagnosis were five and six for females and males, respectively (Table 1). In patients with bladder and renal cancer, most prescriptions were for UTI in females (60.6% and 51.7% for bladder and renal cancer, respectively), and were for obstructive symptoms in males (65.2% and 68.1% for bladder and renal cancer, respectively).

### First prescriptions

In total, 31.8% of females and 44.3% of males had a first prescription for at least one of the three studied ‘chronic’ features (irritative symptoms, obstructive symptoms, and atrophic vaginitis), in the 2 years pre- diagnosis (Table 1). A large proportion of females (39.9%) and males (46.1%) with bladder cancer received a first prescription for irritative and obstructive symptoms, respectively, in the 2 years before diagnosis. This pattern was not seen for renal cancer, with 8.0% and 31.5% of females and males receiving first prescriptions for irritative and obstructive symptoms pre-diagnosis, respectively.

### Rates of prescriptions before diagnosis

**Figure 1.**
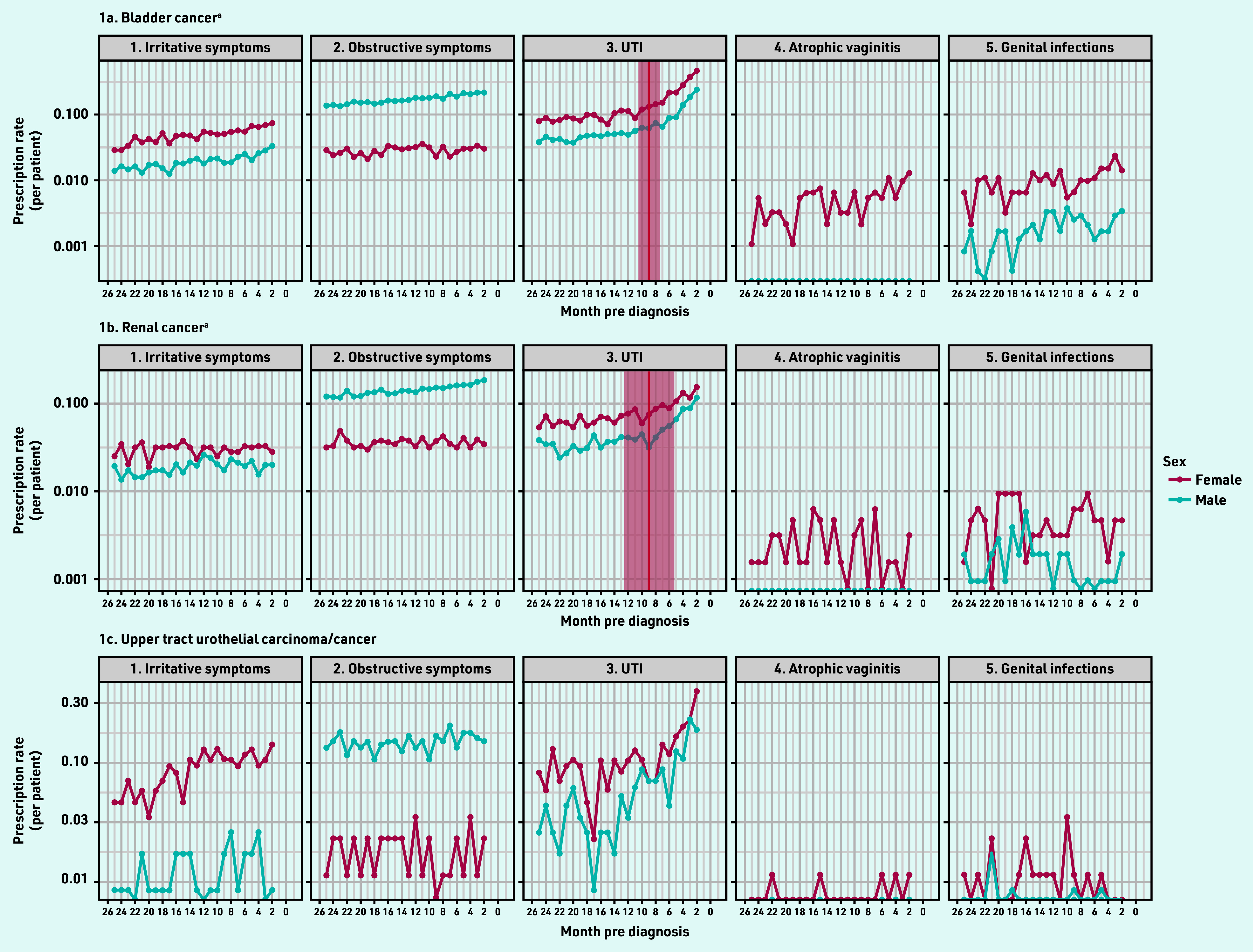
*Rate of prescriptions before diagnosis by cancer site, clinical feature, and sex. ^a^Red vertical line denotes the presence of an estimated inflection point, with the shaded area representing the 95% confidence interval around the estimate (both sexes combined). UTI = urinary tract infection.*

Across all three cancer types, there was an increase in the rate of prescriptions for UTIs in both sexes in the year before diagnosis (Figure 1). In patients with bladder cancer, there was a slight increase in the rate of prescriptions for irritative symptoms in both sexes, for obstructive symptoms in males, and for genital infections and atrophic vaginitis in females in this year. In patients with renal cancer, there was an increase in prescription rate for obstructive symptoms in males.

### Inflection point estimates

Inflection points were identified for UTI prescriptions in bladder and renal cancers, which indicated an increase in the rate of prescriptions above baseline at 9 months for bladder cancer (95% CI = 7.4 to 10.6) and renal cancer (95% CI = 5.3 to 12.7) (Figure 1). Inflection points were identified for alpha-blockers in bladder cancer, and for antimuscarinics, antibiotics, and topical antifungals in UTUC (see Supplementary Figure S1); however, as 95% CIs for these estimates were wide and crossed the 24-month pre-diagnosis point, no pre- diagnostic ‘windows’ could be defined.

**Figure 2.**
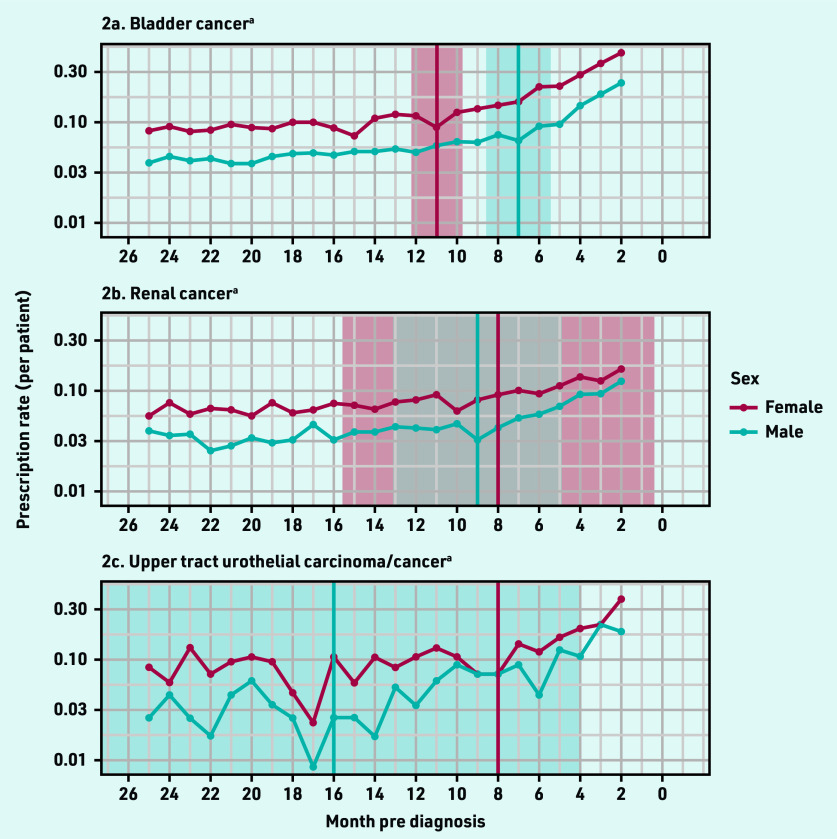
*Antibiotic prescription rates by sex and cancer site. ^a^Red vertical line and red shaded area denote inflection point and 95% confidence interval (CI) for females; blue line and blue shaded area denote inflection point and 95% CI for males; grey shaded area denotes overlapping 95% CI for males and females with renal cancer.*

When examining UTI antibiotic prescriptions by sex for bladder cancer, females had an earlier inflection point at 11 months pre-diagnosis (95% CI = 9.7 to 12.3) compared with 7 months pre- diagnosis (95% CI = 5.4 to 8.6) for males (Figure 2). In patients with renal cancer, there was a smaller difference in the inflection point estimates: males had an earlier infection point than females (9 months, 95% CI = 4.9 to 13.1 versus 8 months, 95% CI = 0.4 to 15.6, respectively).

### Rate of first prescriptions

When first prescriptions were examined for conditions that might be more chronic, there was an increase in the number of alpha- blockers prescribed in male patients with bladder and renal cancer pre- diagnosis, but no pre- diagnostic window could be defined (see Supplementary Figure S2).

## DISCUSSION

### Summary

This study found evidence for an increase from baseline in the rate of prescriptions for UTIs at 9 months before diagnosis of renal and bladder cancer. An earlier inflection point for UTI antibiotic prescriptions was noted in females than in males prior to bladder cancer diagnosis. No pre- diagnostic windows could be defined using prescriptions for other clinical features. The findings demonstrate that some patients are receiving increasing numbers of prescriptions for apparent UTI, which may indicate underlying cancer, many months prior to cancer diagnosis. This indicates that opportunities exist to perform cancer investigations and initiate referrals earlier in the diagnostic window to expedite diagnoses for some patients.

### Strengths and limitations

This study uses a large, routinely collected, representative dataset from primary care and the cancer registry. Important strengths are that the quality of prescription data held by the CPRD is high as prescriptions are not subject to manual coding, and that cancer registry data — which reports high levels of case ascertainment and diagnosis date accuracy — was used to confirm study participants and cancer diagnosis dates for linked cases; this comprised approximately half of the cohort.

Although only patients with confirmed cancer were included, using 2 years’ data allowed for the estimation of inflection points relative to a baseline trend without data from controls who did not have cancer. Bootstrapping allowed for the estimation of 95% CIs, which are more informative than point estimates alone. This direct maximum likelihood method is less prone to bias than similar methods when examining changes in rates of clinical events.^19^ For the purposes of this study, having controls would have added little other than to account for general trends in prescribing in the primary care population over time.

It is important to note that data were used from patients diagnosed up to December 2015. Prescribing practices may have changed since then, particularly during the COVID-19 pandemic period, and further research would be needed to examine the impact of this on inflection points. However, as prescriptions were grouped by clinical feature, changes in the types of medication prescribed by GPs (for example, one antibiotic becoming more widely used than another) would not affect the results.

A further potential limitation of this study is that it was not possible to determine the exact indication for each prescription or whether the medications prescribed were taken by patients. However, the aim was to examine trends in prescribing, rather than reasons for prescriptions or medication concordance.

### Comparison with existing literature

A recent systematic review identified 28 studies exploring different aspects of clinical activity in primary care prior to cancer diagnosis.^7^ Four of these studies examined prescription patterns: two focused on colorectal cancer, one on lung cancer, and one on all cancers. These studies demonstrated that prescriptions for a range of medications increased prior to diagnosis. The findings presented in the current study provide evidence that prescription rates also increase prior to bladder and renal cancer diagnoses, but indicate that these changes only appear to occur for some medication types — notably, antibiotics for UTI. The period prior to diagnosis over which significantly increased prescription rates were detected in previous studies ranged from 6 to 18 months;^7^ this is comparable to the findings presented here, for which changes were noted at 9 months for UTI prescriptions.

The finding that prescriptions for UTI antibiotics increase in the year before diagnosis is in line with current evidence indicating that opportunities exist to expedite diagnosis in patients with UTIs. In particular, the rate of increase of UTI antibiotic prescriptions started earlier in females than males with bladder cancer; this suggests that the propensity to improve diagnostic timeliness is greater in females, and further substantiates current evidence that indicates that females with bladder cancer experience longer diagnostic intervals than males,^20^^,^^22^^–^24 and that they are more likely to receive multiple courses of antibiotics for UTIs before cancer diagnosis than males.^25^

Although some cases may represent genuine concomitant UTI and bladder cancer, the increased use of UTI antibiotics may explain some of the observed delay in referral found in females in other studies.^6^^,^^21^^,^^26^

### Implications for research and practice

The finding that prescribing for UTIs increases in the year prior to bladder and renal cancer diagnosis provides evidence that there is an opportunity to improve diagnostic timeliness in some patients presenting with UTIs, more so in females than males.

Findings substantiate evidence that the sex inequality seen in diagnostic timeliness mainly relate to bladder cancer. This may represent a genuine increase in the frequency of UTI episodes pre- diagnosis (as indicated by the increase in UTI prescriptions) and/or an increase in the symptomatic presentation of bladder cancer mimicking UTI symptoms. The relatively high percentages of patients with bladder cancer in this study, who were given a first prescription for irritative symptoms (females) and obstructive symptoms (males) suggests that, besides UTI, bladder cancer may present with other symptoms, and may present differently in males and females. Further clinical examinations and investigations may therefore be indicated in these patients presenting with irritative or obstructive symptoms.

The observed sex inequality in prescribing patterns in the study presented here can be related to:

- clinicians’ perceptions that many urological symptoms in females may be due to UTIs (given their commonality in females);
- clinicians’ higher threshold for referring females, due to their known lower risk of bladder cancer than males; and
- preference to use antibiotics as a ‘trial of treatment’ for some symptoms.

Furthermore, current referral guidelines from the National Institute for Health and Care Excellence^27^ on suspected cancer recommend that clinicians consider a routine specialist referral in patients with recurrent or persistent UTIs. The lack of a definitive number of UTI episodes and firm recommended action by the guidelines may further contribute to inconsistent clinical practice.^28^ Further research is needed to examine the cancer risk in patients with recurrent UTIs, and identify the higher- risk subpopulation that may benefit from onward referrals, in order to provide evidence for guideline refinement.

Besides UTI prescriptions, there was a slight increase in first prescriptions of alpha- blockers in male patients with bladder and renal cancer, suggesting that males might be presenting more with obstructive or lower urinary tract symptoms in the year before diagnosis. Further research confirming the concordance between prescriptions and clinical codes could shed light on the true indication of these prescriptions.

It is possible that changes in prescription patterns, such as an increase in the frequency of antibiotics for UTI symptoms, could act as an alert for GPs to consider bladder, renal, and UTUC malignancies in individual patients. Further research is needed to determine the predictive value of such changes both alone and alongside other predictors of undiagnosed cancer. Future research should also examine the predictive value of increases in UTI antibiotic prescriptions and the value of different risk-stratifying strategies for improving management of UTIs in patients at risk of urological cancer.

## Data

All data used in this study are available from the CPRD (https://cprd.com). Permission to access the data can be obtained through the ISAC.
